# In vitro *and* in silico anti-dengue activity of compounds obtained from *Psidium guajava* through bioprospecting

**DOI:** 10.1186/s12906-019-2695-1

**Published:** 2019-11-06

**Authors:** Andrea Isabel Trujillo-Correa, Diana Carolina Quintero-Gil, Fredyc Diaz-Castillo, Winston Quiñones, Sara M. Robledo, Marlen Martinez-Gutierrez

**Affiliations:** 1 Grupo de Investigación en Ciencias Animales-GRICA. Facultad de Medicina Veterinaria y Zootecnia, Universidad Cooperativa de Colombia, Bucaramanga, Calle 30A # 33-51, Bucaramanga, Colombia; 2 Programa de Estudio y Control de Enfermedades Tropicales-PECET, Universidad de Antioquia, Calle 70 No. 52–21, Medellín A, A 1226 Colombia; 3 Laboratorio de Investigaciones Fitoquímicas y Farmacológicas de la Universidad de Cartagena – LIFFUC, Universidad de Cartagena, Cartagena, Colombia; 4 Química Orgánica de Productos Naturales, Universidad de Antioquia, Calle 70 No. 52–21, Medellín A, A 1226 Colombia

**Keywords:** Dengue virus, Antiviral, Bioprospecting, *Psidium guajava*, Catechin, Gallic acid, Quercetin

## Abstract

**Background:**

For decades, bioprospecting has proven to be useful for the identification of compounds with pharmacological potential. Considering the great diversity of Colombian plants and the serious worldwide public health problem of dengue—a disease caused by the dengue virus (DENV)—in the present study, we evaluated the anti-DENV effects of 12 ethanolic extracts derived from plants collected in the Colombian Caribbean coast, and 5 fractions and 5 compounds derived from *Psidium guajava*.

**Methods:**

The cytotoxicity and antiviral effect of 12 ethanolic extracts derived from plants collected in the Colombian Caribbean coast was evaluated in epithelial VERO cells. Five fractions were obtained by open column chromatography from the ethanolic extract with the highest selectivity index (SI) (derived from *P. guajava*, SI: 128.2). From the fraction with the highest selectivity (*Pg*-YP-I-22C, SI: 35.5), five compounds were identified by one- and two-dimensional nuclear magnetic resonance spectroscopy. The antiviral effect in vitro of the fractions and compounds was evaluated by different experimental strategies (Pre- and post-treatment) using non-toxic concentrations calculated by MTT method. The DENV inhibition was evaluated by plate focus assay. The results were analyzed by means of statistical analysis using Student’s t-test. Finally the antiviral effect in Silico was evaluated by molecular docking.

**Results:**

In vitro evaluation of these compounds showed that three of them (gallic acid, quercetin, and catechin) were promising antivirals as they inhibit the production of infectious viral particles via different experimental strategies, with the best antiviral being catechin (100% inhibition with a pre-treatment strategy and 91.8% with a post-treatment strategy). When testing the interactions of these compounds with the viral envelope protein in silico by docking, only naringin and hesperidin had better scores than the theoretical threshold of − 7.0 kcal/mol (− 8.0 kcal/mol and − 8.2 kcal/mol, respectively). All ligands tested except gallic acid showed higher affinity to the NS5 protein than the theoretical threshold.

**Conclusion:**

Even though bioprospecting has recently been replaced by more targeted tools for identifying compounds with pharmacological potential, our results show it is still useful for this purpose. Additionally, combining in vitro and in silico evaluations allowed us to identify promising antivirals as well as their possible mechanisms of action.

## Introduction

Dengue is a growing public health problem worldwide, mainly in tropical and subtropical regions [1]. In the last five decades, on the American continent, the incidence of dengue has increased 30 fold and is the cause of approximately 390 million infections per year, of which 96 million have clinical manifestations [2]. In 2013, in America, the largest number of cases in the history of the disease was reported, with a total of 2.3 million cases, an alarming figure [3, 4]. In most cases, dengue presents as an asymptomatic disease, however, it can presented with a wide range of clinical manifestations, including fever, headache, pain in various parts of the body, and prostration, among others. Few patients present with serious life-threatening manifestations. This wide clinical variety permits classified dengue in three groups: asymptomatic patients, symptomatic patients (with or without alarming symptoms) and patients with severe dengue (patients with hemorrhage and/or hypovolemic shock)”) [5].

The etiological agent that causes this disease is the dengue virus (DENV), a member of the *Flaviviridae* family, which belongs to the arboviruses (viruses transmitted by arthropods). This virus is classified into four serotypes (DENV 1–4) according to genetic and antigenic differences [6]. Although any serotype is equally able to cause dengue, serotype differences have been postulated to lead to differences in pathogenesis [7] such as the case for DENV-2 which have been related with sever dengue [8]. The DENV genome consists of a strand of positive-sense Ribonucleic Acid (RNA) of approximately 1 kb with a m7GpppAmp cap at its 5′ end and no poly (A) tail at its 3′-end [9]. Moreover, it has a single reading frame that codes for three structural proteins (C, PrM, and E) and seven nonstructural proteins (NS1, NS2a, NS2b, NS3, NS4a, NS4b, and NS5), which are mainly involved in viral replication [10, 11].

The replication cycle of DENV begins with the E protein binding to receptors on the cell membrane. After this binding, there is a clathrin-mediated endocytosis followed by the formation of an endosome leading to the pH-dependent fusion (viral envelope/endosome). The acidic pH of the endosome favors the release of the viral RNA in the cytoplasm to be transcribed and translated into ribosomes associated to the endoplasmic reticulum, resulting in a single viral polyprotein which is cleaved by cellular and viral proteases. The replication complex (formed by RNA, NS5, other nonstructural proteins and cellular factors) is formed in association with intracellular membranes. Finally, the assembly of viral proteins with new genomes occurs in the lumen of the endoplasmic reticulum, followed by the passage of the new virions through the Golgi apparatus (where the maturation viral process occurs) to be released the virions by exocytosis [12]. Although all steps of the viral replication cycle are likely to be inhibited, most studies have focused on the evaluation of compounds that inhibit the attachment and entry of virus into the cell, among them are heparin [13] and sulfated polysaccharides [13]. Moreover, other compounds are able to inhibit the viral genome replication by blocking the synthesis of nucleoside triphosphates. Ribavirin and mycophenolic acid [14] are two good examples of such inhibitors. Finally, other compounds, including castanospermine [15] and the Lovastatin [16] inhibit steps in the replication cycle after entry and replication of DENV, possibly affecting the assembly process.

Despite the large number of possible antiviral candidates [17], to date, only a few have been tested in clinical trials, such as balapiravir, chloroquine, lovastatin, prednisolone, and celgosivir [18]. However, none of these compounds is being used as an effective anti-dengue therapy. Thus, there remains an important need to identify effective and tolerable medications for treatment of DENV-infected patients both in the early phase, to prevent progression to fatal outcomes, and to minimize deaths after patients develop severe complications [19]. For this reason, the agenda of research priorities proposed by the World Health Organization proposed, a decade ago, that included searching for antivirals (either second-use drugs or natural product derivatives), is still valid today [20]. Moreover, other of the main reasons for the high incidence of the DENV worldwide is that thus far, only one licensed vaccine exists for use in a few countries [21]. This vaccine was produced by Sanofi Pasteur (CYD-TDV) is composed of four attenuated vaccines (CYD-1–4) is a life recombinant vaccine, based on a yellow fever vaccine 17D (YFV 17D) backbone [22].

Plants have been used as medicinal sources to treat many diseases for thousands of years. Ancestral communities could empirically identify plants to fight infections, passing many of these findings from generation to generation until today [23]. Ethnobotany has proven useful for preserving such knowledge in communities that use plants to treat diseases has encouraged bioprospecting studies, which allow the identification of compounds with pharmacological potential [24]. However, is important highlight that in some cases the communities, without scientific knowledge, use plants included in the IUCN Red List of Threatened Species [25] to treat diseases, and for these reason is important encourage to preserve, protect and promote the traditional knowledge but with scientific support.

Due to the diversity of chemical compounds present in plants, they are an important source of pharmacological candidates. Several studies have identified potential plant derived candidates compounds as a potential of new drugs candidates that inhibit the activity of viruses such as herpes virus [26], hepatitis C virus, [27], human immunodeficiency virus type I [28], rotavirus [29], influenza virus, [30], chikungunya virus [31], and DENV [32]. Specifically, for DENV, the antiviral effects of some compounds has been demonstrated, inhibiting both, infectivity and/or viral spread in vitro and in vivo, such as glabranine (derived from *Tephrosia madrensis*) [33], panduratin (derived from *Boesenbergia rotunda* L.) [34], and castanospermine (derived from *Castanospermum australe*) [15]. Recently, inhibitory activity against DENV-2 infection has been identified in ethanolic extracts of Colombian plants such as *Cassia grandis* and *Tabernaemontana cymosa (T. cymosa)*, with 99.9% inhibition observed for the *T. cymosa* extract [35]. Moreover, several compounds derived from *Mammea americana* (coumarins) and *T. cymosa* (lupeol and voacangine) that can inhibit infection in vitro at percentages greater than 50% have been reported [36].

Given the great variety of Colombian plants, the present study evaluated the anti-DENV effect of 12 ethanolic extracts obtained from selected plants based on an ethnobotanical survey conducted in the city of Cartagena (in the Colombian Caribbean coast). The ethanolic extract with the highest selectivity (derived from *Psidium guajava*) was fractionated, and the anti-DENV effect of each fraction was tested. Moreover the results of in vitro assay were contrasted with the in silico assays to postulate promising antivirals.

## Methodology

### Plant selection

Vegetal material was selected based on data obtained from an ethnobotanical survey conducted in the city of Cartagena (Colombia) in 2009 (unpublished results) and from a literature search on plant extracts with reported antiviral activity against viruses that cause febrile illness. In total twelve plants were included: *Ambrosia cumanensis* Kunt (*A. cumanensis*), *Cavanillesia platanifolia* Bonpl (*C. platanifolia*), *Chenopodium ambrosioides* L. (*C. ambrosioides*), *Chrysobalanus icaco* L. (*C. icaco*), *Croton malambo* Karst (*C. malambo*), *Cymbopogon citratus* Stapf (*C. citratus*), *Diospyros inconstans* Jacq (*D. inconstans*), *Mammea americana* L. (*M. americana*), *Momordica charantia* L. (*M. charantia*), *Psidium guajava* L. (*P. guajava*)*, Sarcostemma clausum* Jacq (*S. clausum*), and *Trichilia hirta* L. (*T. hirta*), that were collected between 2009 and 2016 in Cartagena city (Colombia, South America). The collection of most plants was done with the permission of the CARDIQUE (Corporacion Autonoma Regional del Canal del Dique, resolution 0751. June 27/2014). Moreover, the compounds derived from *Psidium guajava* are subject of the contract for access to genetic resources and derived products No. 130 of 2016 (RGE0176) signed with the Ministry of Environment and Sustainable Development of the Republic of Colombia.

### Obtaining extracts and fractions

**Fig. 1:**
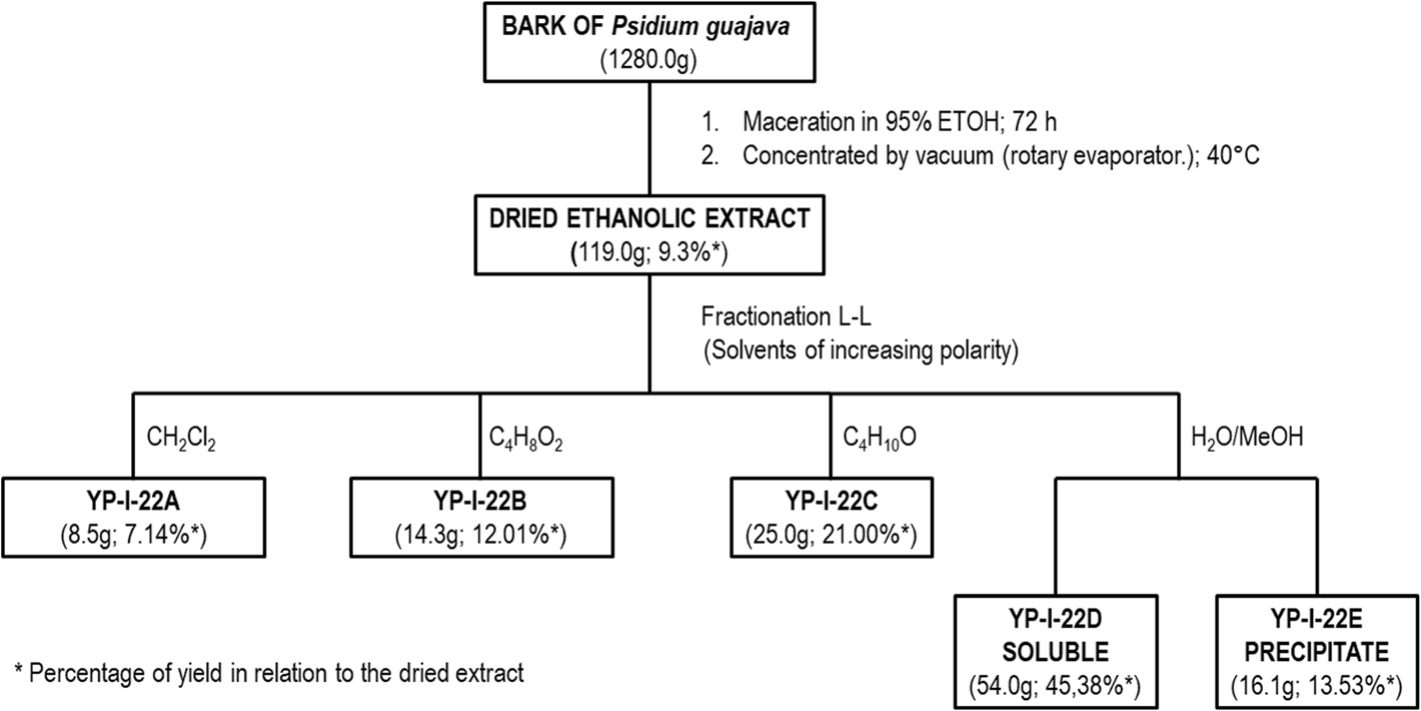
Fractionation of the ethanolic extract of *P. guajava* bark. Open column chromatographic fractionation of the active fraction *Pg*-YP-I-22C obtained from the total ethanol extract of *P. guajava* bark using Sephadex G10 as a stationary phase

Weighed amounts (1000 g) of each plant material were collected for testing (phytochemical and biological examinations) and for identification at the herbarium of the Universidad de Antioquia (HUA) (Medellín, Colombia) and at the herbarium of the Universidad Nacional de Colombia (COL) (Bogotá, Colombia). Moreover, the material was identified by authorized personnel at the Jardín Botanico Guillermo Piñeres in Cartagena (JBC), Colombia (voucher number JBC 1209). The collected plant material was dried at room temperature, weighed, ground, and macerated with 95% ethanol for 72 h. Each sample was then filtered and concentrated in a rotary evaporator. Each dried extract was suspended in a mixture of ethanol-distilled water and subjected to liquid-liquid partitioning with solvents of increasing polarity in the following order: dichloromethane, ethyl acetate, and butanol, as shown in Fig. 1. For the preliminary phytochemical screening of each extract, identification tests were performed for different secondary metabolites according to the method described in [37].

### Isolation and purification of the compounds present in the active fractions

The fractions were subjected to several column chromatographic procedures using silica gel and Sephadex and to normal-phase preparative thin-layer chromatography (PTLC), depending on their polarities, molecular size, and complexity. Final purification was performed using normal or reverse-phase high-performance liquid chromatography (HPLC) as necessary. The structures of the compounds present in the most active fraction of *P. guajava* (*Pg*-YP-I-22C) were elucidated using standard analytical methods, including melting point determination, co-chromatography with reference compounds, and ^1^H nuclear magnetic resonance (NMR) and ^13^C NMR spectroscopy techniques in one and two dimensions, such as the attached proton test (APT), distortionless enhancement of polarization transfer (DEPT), correlation spectroscopy (COSY), nuclear Overhauser effect spectroscopy (NOESY), heteronuclear multiple-quantum correlation spectroscopy (HMQC), and heteronuclear multiple-bond correlation spectroscopy (HMBC).

### Virus and cell maintenance

Epithelial VERO cells (*Cercopithecus aethiops*) were obtained from the American Type Culture Collection (ATCC), and C6/36HT cells (from *Aedes albopictus* mosquito larvae) were donated by Dr. Guadalupe Guzmán, Department of Virology, Pedro Kouri Institute (Havana, Cuba). C6/36HT cells were used to produce the viral stocks and the VERO cells were used to made the antiviral assays.

The cells and the virus strain were maintained according to the protocols described previously [38] . Briefly, the VERO cells were maintained in Dulbecco’s Modified Eagle’s medium (DMEM; Gibco®) supplemented with 2% fetal calf serum (FCS, Gibco®) and were incubated at 37 °C in a 5% CO_2_ atm. The C6/36HT cells were maintained in DMEM (Gibco®) supplemented with 10% FCS (Gibco®) at 34 °C. The DENV 2/NG strain, which was used for all biological assays, was donated by Dr. Jorge Osorio, Department of Pathobiological Sciences, University of Wisconsin (Madison, WI, United States).

### Determination of the selectivity index (SI)

The SI of each ethanolic extract, fraction, or compound was calculated from the relationship between the cytotoxic concentration 50% (CC_50_) and effective concentration 50% (EC_50_) [39]. The CC_50_ was determined via the 3-(4,5-dimethyl-2-thiazolyl)-2,5-diphenyl-2H-tetrazolium bromide) (MTT, Sigma-Aldrich) method. For this, 2.5 × 10^4^ VERO cells were seeded in 96-well plates for 24 h. Subsequently, serial dilutions of the ethanolic extracts (7.8 μg/mL to 1000 μg/mL), fractions (6.25 to 400 μg/mL), and characterized compounds (6.25 to 400 μg/mL) were performed and incubated with the cells for 48 h. After the incubation period, an MTT solution (0.5 mg/mL) was added to the cultures, which were then incubated for an additional 3 h at 37 °C. Finally, dimethyl sulfoxide was added, and the absorbance was read at 450 nm in a Bio-Rad Benchmark® microplate reader. Cultures without extract, fractions, or compounds were processed as positive controls for viability. The CC_50_ was calculated as the extract concentration that reduced cell viability by 50% using regression analysis (Probit software). Each experimental condition was tested in triplicate in two independent experiments (*n* = 6). To calculate the EC_50_, 2.5 × 10^4^ VERO cells were seeded in 96-well plates for 24 h. At 24 h, serial dilutions of each ethanolic extract (7.8 μg/mL to 1000 μg/mL), fraction (6.25 to 400 μg/mL), and characterized compound (6.25 to 400 μg/mL) were mixed with the DENV-2/NG strain (at a multiplicity of infection (MOI = 1) and added to the cell monolayers for 2 h. At 2 h post-inoculation (hpi), the mixtures were removed, and the same serial dilutions of the extracts, fractions, or compounds were added again and incubated for 24 h. Finally, the supernatants were collected and stored at − 70 °C until performing the multiwell plate focus assay [40].

### Multiwell plate focus assay

A total of 2.5 × 10^4^ cells/well were seeded in 96-well plates. The cells were inoculated the following day with serial dilutions (10^− 1^ to 10^− 6^) of the supernatants from the EC_50_ experiments for 2 h. Next, the inoculum were removed, and 1.5% carboxymethylcellulose (Sigma-Aldrich) prepared in DMEM (Gibco®) supplemented with 2% FCS (Gibco®) was added, followed by incubation at 37 °C in a 5% CO _2_ atmosphere. On the third day, the cells were fixed with a methanol-acetone (1:1) solution for 10 min, washed three times with PBS, and permeabilized with 0.1% Triton X-100 for 30 min, after which nonspecific site blocking was performed with 10% FBS in PBS. Then, DENV anti-envelope monoclonal antibody (mAb) was diluted 1:500 with PBS containing 10% FCS (Gibco®) was added, followed by incubation for 1 h at 37 °C. After washing with PBS, the plates were incubated for 30 min with an anti-mouse IgG secondary antibody conjugated to peroxidase. Finally, the reactions were stained with 3-amino-9-ethylcarbazole (AEC, Sigma-Aldrich), and the number of foci in each well were counted.

### Determining the antiviral effects of the fractions and compounds on virus cell entry

A total of 2.5 × 10^4^ VERO cells were seeded in 24-well plates for 24 h, and the fractions (100 μg/mL) or characterized compounds (50 μg/mL) were then added and incubated with the cells for an additional 24 h (pre-treatment strategy) [16]. Subsequently, treatment was withdrawn, and a viral inoculum (DENV-2/NG at an MOI = 1) was added and incubated for 2 h. Subsequently, the virus was removed, and fresh medium was added for an additional 48 h, after which the supernatants were collected and stored at − 70 °C until further processing via the multiwell plate focus assay. For each fraction and compound, two independent experiments were performed each with two replicates (*n* = 4). Heparin (Sigma-Aldrich) served as the positive control for viral inhibition [41].

### Determining the antiviral effects of the fractions and compounds after virus cell entry

A total of 2.5 × 10^4^ VERO cells were seeded in 24-well plates for 24 h, and a viral inoculum (DENV-2/NG at an MOI = 1) was then added and incubated for 2 h. Subsequently, the inoculum was removed, and the fractions (100 μg/mL) or characterized compounds (50 μg/mL) were added and incubated with the cells for an additional 48 h (post-treatment strategy) [16]. Next, the supernatants were collected and stored at − 70 °C until further processing via the multiwell plate focus assay. For each fraction or compound, two independent experiments were performed each with two replicates (*n* = 4). Suramin served as the positive control for viral inhibition [41].

### Viral inhibition assay in VERO cells

A total of 2.5 × 10^4^ VERO cells were seeded in 24-well plates, and a viral inoculum was added at 24 h, followed by incubation for 2 h. Subsequently, the inoculum was removed, and the cells were treated with a single noncytotoxic concentration of each of the five fractions and compounds obtained from the fraction with the highest selectivity. At 48 h, the supernatants were removed and stored at − 70 °C until further processing via the multiwell plate focus assay. Suramin served as the positive control for viral inhibition [41].

### Indirect immunofluorescence

Some cell monolayers were washed with Phosphate Buffered Saline pH 7.4 (PBS, Gibco® and fixed with 3.8% paraformaldehyde (PFA, Sigma-Aldrich) in PBS at 37 °C for 30 min. The cells were then treated with 50 mM NH_4_Cl for 10 min at room temperature and permeabilized with 0.3% Triton X-100, after which the nonspecific sites were blocked with 5% FBS. To detect the E protein, the monolayers were incubated sequentially with an anti-DENV-2 envelope primary mAb and an anti-mouse secondary antibody conjugated to Alexa 488. Finally, the plates were examined using a NIKON Eclipse TS100 microscope.

### Molecular docking

For molecular docking, the structures of the isolated compounds and that of suramin were downloaded from the DrugBank database or were constructed from SMILES strings using CHIMERA [42]. The three-dimensional structures of two viral proteins, the structural envelope protein (E, PDB: 3UZV) and the nonstructural protein 5 (NS5, PDB: 2J7U), were obtained by using the Protein Data Bank (PDB) database. Structures with a resolution equal to or less than 2.5 Å were considered. The 3D models of interest were prepared for docking using the Autodock tools package, removing both the water molecules and co-crystallized molecules not part of the target protein and adding Gasteiger charges and nonpolar hydrogens. To determine the active sites or binding sites of the protein molecules, the CASTp tool was used [43]. According to this, a grid box coordinates were defined as follows: center x = − 23.889, center y = − 4.278, and center z = − 30.778 for E and center x = 22.029, center y = 68.945, and center z = 22.667 for NS5, with both boxes having 1 Å spacing and a number of points in xyz = 30 and being evaluated with an exhaustiveness of 10. Finally, Autodock Vina software (Version 1.1.2) was used to determine the best interactions between the viral proteins and compounds [44], For Autodock Vina running, a configuration file including the name of the protein with the extension *pdbqt, the ligand name with the same extension, the grid box center and the exhaustiveness, was prepared as a CONF file. Best interations were identified based on a score from 0 to − 7.0 kcal/mol [43], with scores equal to or less than − 7.0 kcal/mol corresponding to the best affinities. The generated interactions were analyzed using LigPlot+ v1.4.5 [45].

### Statistical analysis

To determine the CC_50_ and EC_50_, a regression analysis was performed with Probit Software. The SI of each molecule was determined from the relationship between the CC_50_ and EC_50_, using the formula SI = CC_50_/EC_50._ To compare the number of infectious viral particles released between the cells treated with each fraction or compound (for both the pre- and post-treatment strategies) and the untreated cells, Student’s t-test was used. All statistical analyses were performed using the Prism® 7.01 for Windows™ package (GraphPad Software, San Diego, CA), considering differences statistically significant at *p* < 0.05 in all cases. Each experimental condition was tested in triplicate in two independent experiments (*n* = 6) to guaranty the statistical analysis and the figures show the average of those experiments.

## Results

### Antiviral effects of the ethanolic extracts

Overall, the cytotoxicity of the extracts was low (all had CC_50_ values greater than 100 μg/ml). The extract with the highest CC_50_ was derived from the bark of *P. guajava* (CC_50_ = 1000 μg/mL), and the extract with the lowest CC_50_ was from the leaves of *A. cumanensis* (CC_50_ = 112.4 μg/mL). Considering antiviral effectiveness, we found a wide range of effective concentration values. The extract with the highest EC_50_ was derived from the bark of *D. inconstans* (EC_50_ = 538.6 μg/mL), and the extract with the lowest EC_50_ was from the bark of *P. guajava* (EC_50_ = 7.8 μg/mL). Finally, the extracts were classified into four groups based on their SI values (calculated according to the CC_50_/EC_50_ ratio). The first group contained nonselective extracts (SI < 2.0), including those derived from the leaves of *C. citratus* (SI = 0.5), the leaves of *S. clausum* (SI = 1.2), the bark of *D. inconstans* (SI = 1.4), and the seeds of *C. icaco* (SI = 1.7). The second group comprised extracts with low selectivity (SI ≥ 2.0 and < 5), including those from the seeds of *T. hirta* (SI = 3.4), the bark of *C. malambo* Karst (SI = 4.1), and the leaves of *A. cumanensis* Kunt (SI = 4.2). The third group contained extracts with moderate selectivity (SI ≥ 5 and < 10), including those from the leaves of *C. ambrosioides* (SI = 5.3), the fruit of *C. platanifolia* Bonpl (SI = 5.8), the seeds of *M. charantia* (SI = 7.9), and the leaves of *M. americana* (SI = 8.4). Finally, the group with high selectivity (SI ≥ 10) included the extract from the bark of *P. guajava* (SI = 128.2). Based on these results (Table 1), the bark extract of *P. guajava* was further investigated via fractionation*.*

**Table 1:** Cytotoxic concentration 50% (CC_50)_, effective concentration 50% (EC_50)_, and selectivity index **(**SI) values of the evaluated ethanolic extracts, fractions, and compounds in VERO cells infected with DENV-2/NG

Type of compound	Scientific name	Family	Voucher number	Plant part	CC_50_	CE_50_	SI
Ethanolic extracts	*Cymbopogon citratos Staf*	Poaceae	*JBC 12015*	Leaves	155.1	343.1	0.5
	*Sarcostemma clausum Jacq*	Asclepiadaceae	*JBC 2502*	Leaves	565.7	458.7	1.2
	*Diospyros inconstans Jacq*	Ebenaceae	*JBC 1438*	Bark	727.1	538.6	1.4
	*Chrysobalanus icaco L*	Chrysobalanaceae	*JBC 934*	Seeds	550.8	325.8	1.7
	*Trichilia hirta L*	Meliaceae	*JBC 917*	Seeds	213.6	62.9	3.4
	*Croton malambo Karst*	Euphorbiaceae	*JBC 12008*	Bark	127.3	31.3	4.1
	*Ambrosia cumanensis Kunt*	Asteraceae	*COL 538448*	Leaves	112.4	26.7	4.2
	*Chenopodium ambrosioides L*	Chenopodiaceae	*JBC 4005*	Leaves	131.6	24.8	5.3
	*Cavanillesia platanifolia Bonpl*	Bombacaceae	*JBC 47576*	Almond	252.6	43.5	5.8
	*Momordica charantia L*	Cucurbitaceae	*JBC 793*	Seeds	125.3	15.9	7.9
	*Mammea americana L*	Calophyllaceae	*JBC 467*	Leaves	440.7	52.3	8.4
	*Psidium guajava L*	Myrtaceae	*JBC 1209*	Bark	1000.0	7.8	128.2
Fractions	*Psidium guajava L*	–	*HUA 140931*	*Pg*-YP-I-22A	130.5	134.4	1.0
				*Pg*-YP-I-22B	308.9	26.5	11.7
				*Pg*-YP-I-22C	625.7	17.7	35.4
				*Pg*-YP-I-22D	177.9	56.1	3.2
				*Pg*-YP-I-22E	102.2	16.7	6.1
Compounds	*Pg*-YP-I-22C	–	–	Gallic Acid	543.4	25.8	21.1
				Naringin	646.8	47.9	13.5
				Quercetin	659.8	19.2	34.3
				Catechin	833.3	33.7	24.8
				Hesperidin	413.8	225.8	1.8

Group A: No selectivity, SI < 2.0

Group B: Low selectivity, SI ≥ 2.0 and < 5

Group C: Moderate selectivity, SI ≥ 5 and < 10

Group D: High selectivity, SI ≥ 10

• The plants *Cymbopogon citratos Staf, Sarcostemma clausum Jacq, Diospyros inconstans Jacq, Chrysobalanus icaco L, Trichilia hirta L and Croton malambo Karst, Chenopodium ambrosioides L, Cavanillesia platanifolia Bonpl, Momordica charantia L, Mammea americana L, Psidium guajava L* were identified at the Jardin Botanico de Cartagena (JBC)

• The plant *Ambrosia cumanensis Kunt* was identified at the Herbarium of the Universidad Nacional de Colombia (COL)

• The plant used to obtain the fractions from *Psidium guajava L* was identified at the Herbarium of the Universidad de Antioquia (HUA)

### Preliminary phytochemical screening of the *Psidium guajava* bark fractions

From the bark extract of *P. guajava*, five fractions were obtained: *Pg*-YP-I-22A, *Pg*-YP-I-22B, *Pg*-YP-I-22C, *Pg*-YP-I-22D, and *Pg*-YP-I-22E. Phytochemical characterization showed that alkaloids and tannins were present in all fractions except for *Pg-*YP-I-22A. Flavonoids were found in *Pg*-YP-I-22C, *Pg*-YP-I-22D, and *Pg*-YP-I-22E; glycosides in *Pg*-YP-I-22C and *Pg*-YP-I-22D; triterpenes in *Pg*-YP-I-22A and *Pg*-YP-I-22B; and sterols in *Pg*-YP-I-22A, *Pg*-YP-I-22B, and *Pg*-YP-I-22C. Finally, coumarins were detected only in *Pg-*YP-I-22D, and saponins or quinones were not detected in any of the fractions. Table 2 shows these results.

**Table 2:** Phytochemical screening of the *P. guajava* bark fractions

Metabolite	*Pg*-YP-22A	*Pg*-YP-22B	*Pg*-YP-22C	*Pg*-YP-22D	*Pg*-YP-22E
Alkaloids	–	+++	+++	+++	+++
Tannins	–	+	+++	+++	+++
Flavonoids	–	–	+++	++	++
Glycosides	–	–	+	++	–
Triterpenes	++	++	–	–	–
Sterols	++	++	+	–	–
Coumarins	–	–	–	+	–
Saponins	–	–	–	–	–
Quinones	–	–	–	–	–

### Antiviral effects of the fractions obtained from the *P. guajava* extract

**Fig. 2:**
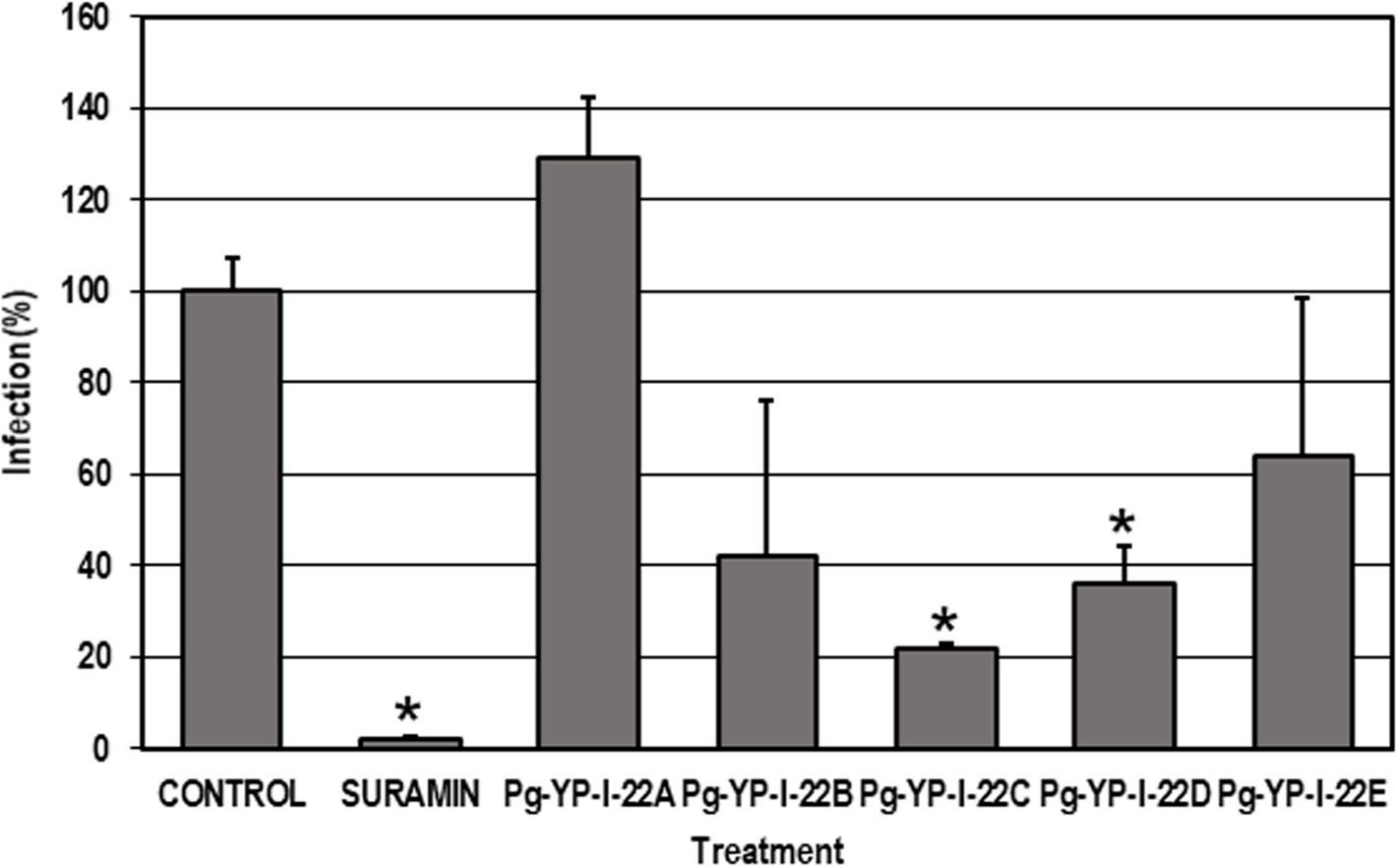
Inhibition of infectious particle production by the *P. guajava* fraction. Cultures were infected (MOI = 1) and then treated for 48 h with each of the five fractions (100 μg/mL). The error bars correspond to the SEM. The asterisks indicate cases with statistically significant differences (Student’s t-test; *p* < 0.05) in relation to the control without treatment. N = 4

Overall, the cytotoxicity of the fractions was low with CC_50_ values greater than 100 μg/mL,the least toxic fraction was *Pg*-YP-I-22C (CC_50_ = 625.7 μg/mL). Considering anti-DENV effectiveness, we found that most fractions were effective at low concentrations (EC_50_ values of less than 100 μg/mL) except for *Pg*-YP-I-22A (EC_50_ = 134.4 μg/mL). Finally, SI values were calculated for the fractions, which were then classified into the same four groups as those for the extracts. Accordingly, *Pg*-YP-I-22A was nonselective (SI = 1.0), *Pg*- YP-I-22D had low selectivity (SI = 3.2), *Pg*-YP-I-22E was moderately selective (SI = 6.1), and *Pg*-YP-I-22B and *Pg*-YP-I-22C were highly selective (SI = 11.7 and 35.4, respectively). Table 1 shows these results. Considering the cytotoxicity results, a nontoxic concentration (100 μg/mL) was chosen to evaluate the antiviral effects of said fractions via an inhibition test, which examined the production of infectious viral particles (Fig. 2). Only *Pg*-YP-I-22C and *Pg*-YP-I-22D significantly inhibited infection compared to the control without treatment (*p* < 0.05; the inhibition rates were 78.2 and 63.7%, respectively). After evaluating the SI values and virus replication inhibition percentages, *Pg*-YP-I-22C was chosen for further testing as its selectivity was high (SI: 35.4) and viral replication inhibition rate of 78.2%.

### Identification of the compounds in the *Pg*-YP-I-22C fraction

**Fig. 3:**
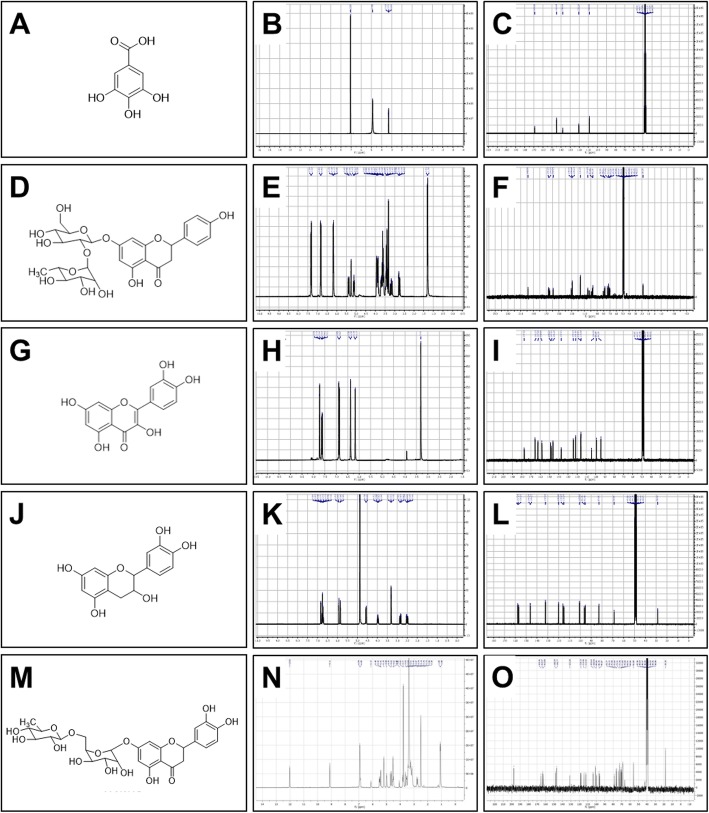
^1^H-NMR- and ^13^C-NMR-derived structures and spectra of the compounds isolated from the *Pg*-YP-I-22C fraction. The five compounds isolated from the *Pg*-YP-I-22C fraction were identified by one- and two-dimensional nuclear magnetic resonance spectroscopy (1D and 2D NMR) techniques and through comparisons with data reported in the literature. **a**, **d**, **g**, **j**, and **m**. Chemical structures. **b**, **e**, **h**, **k** and **n**. Proton spectra of each compound. **c**, **f**, **i**, **l**, and **o**. ^13^C spectra of each compound. **a**-**c**. Gallic acid. **d**-**f**. Naringin. **g**-**i**. Quercetin. **j**-**l**. Catechin. **m**-**o**. Hesperidin

Compounds 22CK001, 22CK002, 22CK003, 22CK004, and 22CK005 were isolated from the *Pg-*YP-I-22C fraction. Table 3 shows the results of the open column chromatography experiments. Next, the compounds were identified by one- and two-dimensional NMR spectroscopy (1D and 2D NMR) techniques and through comparisons with data reported in the literature (Fig. 3).

**Table 3:** Open column chromatographic fractionation of the active fraction *Pg-*YP-I-22C obtained from the ethanolic extract of *P. guajava* bark

Fraction code	Mobile phase^a^	Weight (mg)	Performance (%)
22CK001	MeOH:Acetic acid 0.1% (10:90)	77.5	7.8
22CK002	MeOH:Acetic acid 0.1% (15:85)	17.5	1.8
22CK003	MeOH:Acetic acid 0.1% (20:80)	22.0	2.2
22CK004	MeOH:Acetic acid 0.1% (25:75)	44.5	4.5
22CK005	MeOH:Acetic acid 0.1% (25:75)	27.9	2.8

^a^ Stationary Phase: Sephadex G10. Ratio sample/Sephadex: 1:20

Compound 22CK001 exhibited the following physical and spectral properties: white solid. MP 251 °C. ^1^H NMR (300 MHz, MeOD) δ 7.07 (2H, s, H-2/H-6). ^13^C NMR (75 MHz, MeOD) δ 170.40 (s, COOH), 146.38 (d, C-2/C-6), 139.58 (s, C-1), 121.94 (s. C-4), 110.30 (s, C3/C5). This compound was identified as gallic acid (Fig. 3a) by comparing its ^1^H-NMR and ^13^C-NMR physicochemical and spectral data (Fig. 3b and c, respectively) with those reported in the literature.

Compound 22CK002 exhibited the following physical and spectral properties: amorphous solid. MP 85 °C. ^1^H NMR: δ 1.29 (3H, d, *J* = 6.0 Hz, 5″′-CH_3_), 2.74 (1H, dd, *J* = 18.0, 3.0 Hz, H-3*ax*), 3.14 (1H, dd, *J* = 15.0, 3.0 Hz, H-3 *eq*), 3.06–3.19 (2H, 3.13 (dd, *J* = 10.3, 10.2 Hz), 3.12 (dd, *J* = 10.3, 10.2 Hz)), 3.24–3.34 (2H, 3.26 (dd, *J* = 3.5, 2.9 Hz), 3.31 (dd, *J* = 10.2, 3.5 Hz)), 3.35–3.50 (3H, 3.41 (dt, *J* = 10.3, 6.5 Hz), 3.42 (t, *J* = 10.2 Hz), 3.45 (dd, *J* = 10.3, 10.2 Hz)), 3.80–3.84 (2H, 3.82 (d, *J* = 6.5 Hz), 3.82 (d, *J* = 6.5 Hz)), 4.03 (1H, dq, *J* = 10.3, 6.7 Hz), 5.10 (1H, d, *J* = 6 Hz, H1″′), 5.25 (1H, bs, H-2″), 5.37 (1H, dd, *J* = 12.0, 3.0 Hz), 6.13 (1H, d, *J* = 3.0 Hz, H-8), 6.15 (1H, d, *J* = 3.0 Hz, H-6), 6.83 (2H, d, *J* = 9.0 Hz, H-3′/ H-5′), 7.31 (2H, d, *J* = 9.0 Hz, H-2′/ H-6′). ^13^C NMR (75 MHz, MeOD) δ 198.54 (s, C-4), 166.58 (s, C-7), 166.50, 165.00, 164.95, 164.66, 164.63, 159.12, 130.76, 129.18, 116.34, 104.89, 102.58, 102.51, 99.36, 99.31, 97.85, 96.74, 80.72, 79.17, 79.02, 78.95, 78.11, 73.90, 72.15, 71.21, 69.98, 64.73, 62.25, 43.95 (t, C-3), 18.22 (q, C-6). This compound was identified as naringin (Fig. 3d) by comparing its ^1^H-NMR and ^13^C-NMR physicochemical and spectral data (Fig. 3e and f, respectively) with those reported in the literature.

Compound 22CK003 exhibited the following physical and spectral properties: yellow needles (from methanol). MP 316 °C. ^1^H NMR [300 MHz, CD_3_OD, δ (ppm)]: ^1^H NMR (300 MHz, MeOD) δ 7.73 (d, *J* = 3.0 Hz, H-2′), 7.61 (dd, *J* = 3.0 Hz, 9.0 Hz, H-6′), 6.88 (d, *J* = 8.4 Hz, H-5′), 6.38 (d, *J* = 3.0 Hz, H-8), 6.18 (d, *J* = 3.0 Hz, H-6). ^13^C NMR (75 MHz, CD_3_OD) δ 177.27 (C-4); 165.51 (C-7), 162.45 (C-5), 158.17 (C-2), 148.71 (C-4′), 147.93 (C-3′), 137.18 (C-3), 124.10 (C-6′), 121.64 (C-1′), 116.18 (C-2′), 115.95 (C-5′), 104.48 (C-10), 99.19 (C-6), 94.37 (C-8). This compound was identified as quercetin (Fig. 3g) by comparing its ^1^H-NMR and ^13^C-NMR physicochemical and spectral data (Fig. 3h and i, respectively) with those reported in the literature.

Compound 22CK004 exhibited the following physical and spectral properties: Orange amorphous solid. 213 °C. ^1^H NMR (300 MHz, CD_3_OD) δ 4.59 (d, *J* = 6.0 Hz, H-2), 4.01 (ddd, *J* = 9.0, 6.0, 6.0 Hz, H-3), 2.52 (dd, *J* = 15.0, 6.0 Hz, H-4b), 2.84 (dd, *J* = 15.0, 6.0 Hz, H-4a), 5.93 (d, *J* = 3.0 Hz, H-6), 5.85 (d, *J* = 3.0 Hz, H-8), 6.85 (d, *J* = 1.5 Hz, H-2′), 6.79 (d, *J* = 9.0 Hz, H-5′), 6.72 (dd, *J* = 1.5, 9.0 Hz, H-6′). ^13^C NMR (75 MHz, CD_3_OD) δ 82.85 (C-2), 68.81 (C-3), 28.52 (C-4), 157.58 (C-5), 96.26 (C-6), 157.83 (C-7), 95.48 (C-8), 156.91 (C-9), 100.80 (C-10), 132.20 (C-1′), 115.24 (C-2′), 146.24 (C-3′), 146.22 (C-4′), 116.07 (C-5′), 120.04 (C-6′). This compound was identified as catechin (Fig. 3j) by comparing its ^1^H-NMR and ^13^C-NMR physicochemical and spectral data (Fig. 3k and l, respectively) with those reported in the literature.

Compound 22CK005 exhibited the following physical and spectral properties: amorphous solid. MP 250–253 °C. ^1^H NMR (DMSO-d6, 300 MHz) δ 12.02 (1H, br s, 5-OH), 6.93 (1H, d, *J* = 2.0 Hz, H-2′), 6.86 (1H, *J* = 8.0 Hz, H-5′), 6.83 (1H, dd, *J* = 8.0, 2.0 Hz, H-6′), 6.13 (1H, d, *J* = 2.0 Hz, H-8), 6.11 (1H, d, *J* = 2.0 Hz, H-6), 5.43 (1H, dd, *J* = 11.0, 5.0 Hz, H-2), 4.96 (1H, d, *J* = 7.2 Hz, H-1″), 4.54 (1H, br s, H-1), 3.80 (3H, s, 4-OCH3), 3.20–3.63 (6H, m, H-2″ to H-6″), 3.20–3.63 (3H, m, H-2 to H-6), 3.08 (1H, dd, *J* = 17.0, 11.0 Hz, H-3a), 2.74 (1H, dd, *J* = 17.0, 5.0 Hz, H-3b), 2.48 (1H, d, *J* = 6.0 Hz, H-5), 1.06 (3H, d, *J* = 6.0 Hz, H-6); ^13^C NMR (DMSO-d6, 75 MHz) δ 197.4 (s, C-4), 165.2 (s, C-7), 163.0 (s, C-5), 162.7 (s, C-9), 147.8 (s, C-4′), 146.2 (s, C-3′), 131.0 (s, C- 1′), 117.9 (s, C-6′), 114.2 (d, C-2′), 112.0 (d, C-5′), 103.1 (s, C-10), 100.7 (d, C-1), 99.3 (d, C-1″), 96.1 (d, C-6), 95.4 (d, C-8), 78.3 (d, C-2), 76.4 (d, C-5″), 75.4 (d, C-3″), 73.0 (d, C-4), 72.3 (d, C-2″), 71.02 (d, C-4″), 70.3 (d, C-3), 69.3 (d, C-2), 68.6 (d, C-5), 66.4 (t, C-6″), 55.5 (q, 4-OCH3), 42.4 (t, C-3), 18.12 (q, C-6). This compound was identified as hesperidin (Fig. 3m) by comparing its ^1^H-NMR and ^13^C-NMR physicochemical and spectral data (Fig. 3n and o) with those reported in the literature.

### Anti-DENV effects of the compounds obtained from the *Pg*-YP-I-22C fraction

The toxicity of the compounds was very low with CC_50_ values greater than 400 μg/mL; catechin was the least toxic (CC_50_ = 833.3 μg/mL). For antiviral effectiveness, we found that four of the five compounds were effective at concentrations lower than 100 μg/mL, with the most effective being quercetin (EC_50_ = 19.2 μg/mL); hesperidin was effective only at a higher concentration (EC_50_ = 225.8 μg/mL). Finally, the SI was calculated for each compound. Hesperidin was the only compound not considered selective, as it had an SI value less than 2.0 (SI: 1.8); all of the other compounds were considered highly selective, with quercetin being the most selective (SI: 34.3). Table 1 shows these results.

### Anti-DENV effects of the compounds on some steps of the DENV replication cycle

**Fig. 4:**
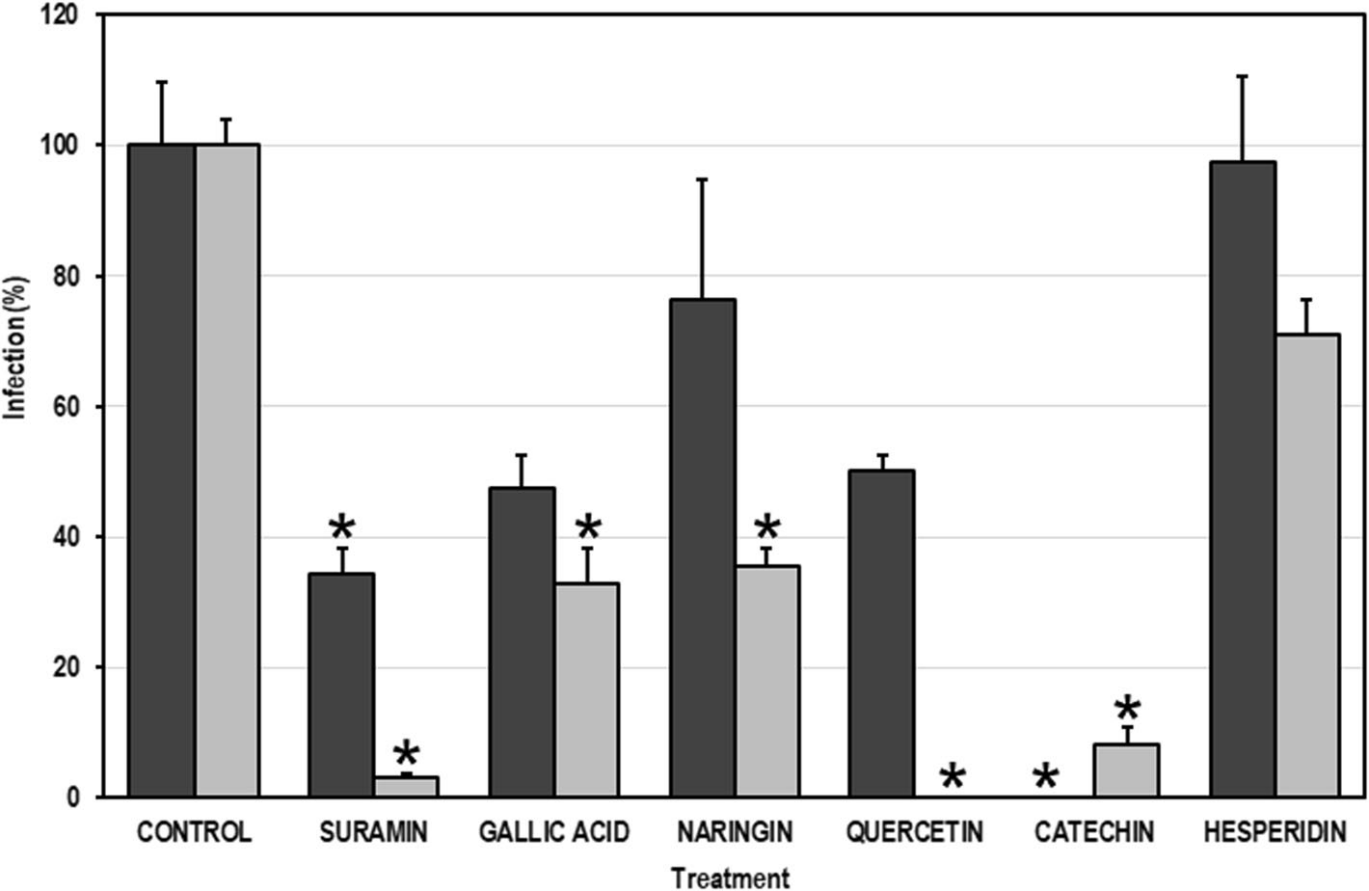
Antiviral effects of the compounds derived from the *Pg-* YP-I-22C fraction on some steps of the DENV replication cycle. The cells were treated with the compounds for 48 h and then infected with DENV (MOI = 1). This infection strategy (pre-treatment) is represented by the dark bars. In addition, independent cell cultures were infected with DENV (MOI = 1) and then treated with the compounds (post-treatment strategy; indicated by clear bars). The error bars correspond to the SEM. The asterisks indicate cases with statistically significant differences (Student’s t-test; *p* < 0.05) in relation to the control without treatment

**Fig. 5:**
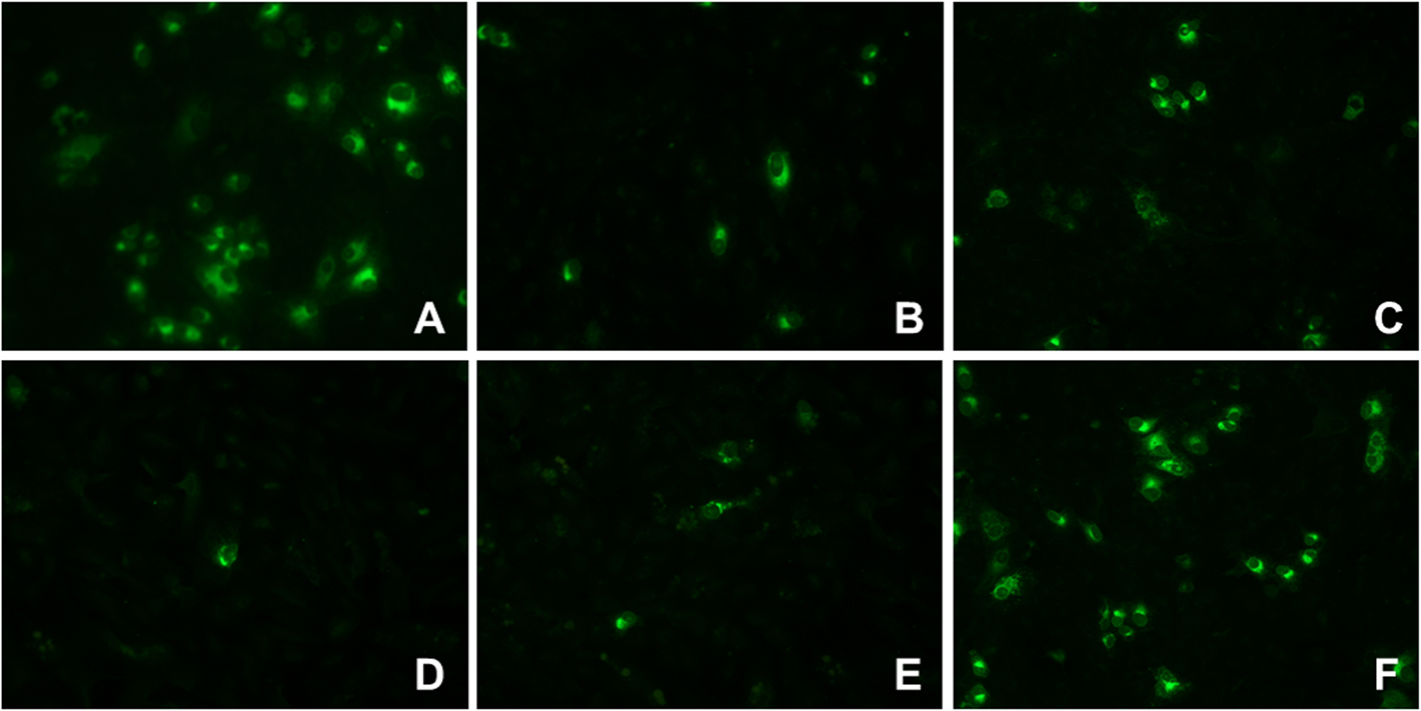
Immunodetection of viral antigen (E Protein) in cultures of VERO cells. Representative images of cultures infected and later treated with each of the compounds isolated from the *Pg*-YP-I-22C fraction. **a**. Control without treatment. **b**. Gallic acid. **c**. Naringin. **d**. Catechin. **e**. Quercetin. **f**. Hesperidin

Using noncytotoxic concentrations of each compound, two different experimental strategies were performed (pre- and post-treatment strategies) to evaluate their effects on processes before or after viral entry. In the pretreatment strategy, only gallic acid, quercetin, and catechin decreased infection in a statistically significant way (with viral inhibition percentages of 52.6, 50.0, and 100%, respectively). Conversely, in the post-treatment strategy, all the compounds except for hesperidin inhibited infection in a statistically significant way; the viral inhibition percentage was highest in the cultures treated with quercetin (100.0%), followed by catechin (91.8%), naringin (64.5%), and gallic acid (67.3%) (Fig. 4). These results were confirmed by decreases in viral antigen in the infected cultures treated with each compound (Fig. 5).

### In silico analysis of binding between the compounds and viral proteins

**Fig. 6:**
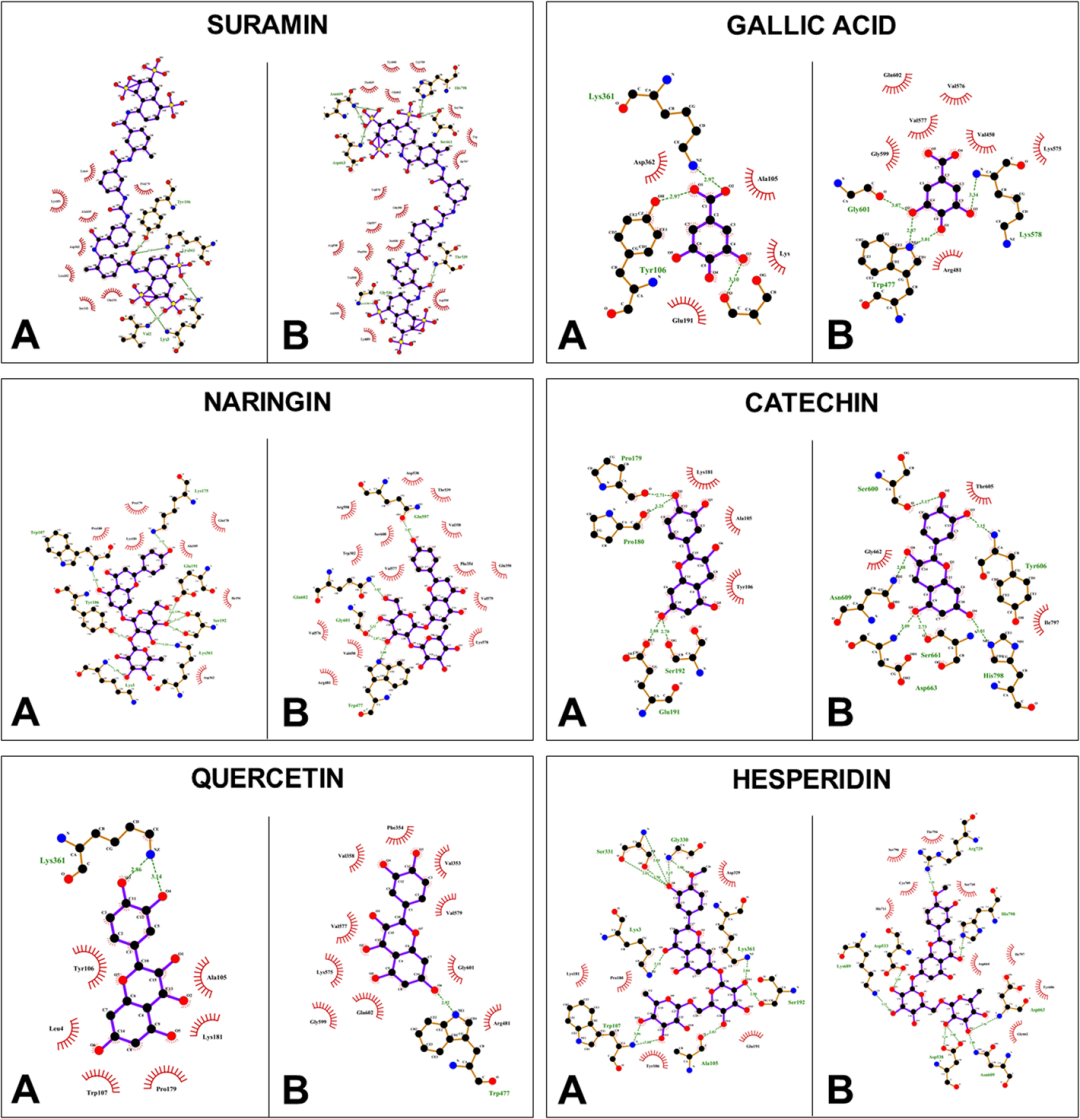
Protein interactions with the compounds isolated from the *Pg*-YP-I-22C fraction. **a**. Interactions of viral Protein E with the compounds. **b**. Interactions of viral Protein NS5 with the compounds. Hydrogen bond interactions are represented by dashed lines in green, and their distances are indicated (Å). Hydrophobic interactions are represented as red eyelashes. The names and numbers in the residues correspond to the target proteins. Images obtained using LigPlot+ v.1.4.5

The docking energy of the five flavonoids and suramin was examined using Autodock Vina. In this study, domain III of the E protein and the polymerase domain of the NS5 protein were used. All ligands showed favorable binding energies, and the interactions with the E protein were mediated by hydrogen bonds in addition to at least one hydrophilic amino acid (Ser192 and/or Lys361); only two of the five compounds, naringin (− 8.0 kcal/mol) and hesperidin (− 8.2 kcal/mol), had better scores than the theoretical threshold of − 7.0 kcal/mol with the E protein (Table 4). The control compound suramin interacted to the E protein with a binding energy of − 7.9 kcal/mol, forming four hydrogen bonds with Val2, Lys3, Tyr106, Lys361, and to the NS5 protein with a binding energy of − 12.0 kcal/mol forming six hydrogen bonds (Fig. 6); all ligands tested except for gallic acid (− 5.3 kcal/mol) had docking energies above the theoretical threshold when interacting with the NS5 protein (Table 4), and their interactions were mediated by 1 to 7 hydrogen bonds. Among the compounds, although the hesperidin-NS5 interaction had the highest number of hydrogen bonds, the shortest distance to a hydrogen bond between the ligand and target protein appeared in the catechin-NS5 interaction (2.73 Å). The amino acids Trp477 and Gln601 participated in the interaction with gallic acid, naringin, and quercetin, while the amino acids Asn609, Asp663, and His798 were involved in the interaction with catechin and hesperidin, as well as suramin (Table 4, Fig. 6).

**Table 4:** Docking scores for the interaction between *P. guajava* compounds and DENV E and NS5 proteins

Target	Compound	Binding energy (Kcal/mol)	Hydrogen bonds	Minimum distance between H bonds (Å)	Residues forming H bonds	Residues participating in hydrophobic interactions
E	SURAMIN	- 7.9	5	2.88	Val2, Lys3, Tyr106, Lys361	Leu4, Pro179, Lys181, Leu182, Glu191, Ser192, Asp362
	GALLIC ACID	- 4.9	3	2.97	Tyr106, Ser192, Lys361	Ala105,Lys181, Glu191, Ile194, Asp362
	NARINGIN	- 8.0	9	2.71	Lys3, Tyr106, Trp107, Lys175, Glu191, Ser192, Lys361	Ala105, Gln178, Pro179, Pro180, Lys181, Ile194, Asp362
	QUERCETIN	- 6.6	2	2.86	Lys361	Leu4, Ala105, Ty106, Trp107, Pro179, Lys181
	CATECHIN	- 6.4	4	2.70	Pro179, Pro180, Glu191, Ser192	Ala105, Tyr106, Lys181
	HESPERIDIN	- 8.2	11	2.83	Lys3, Ala105, Trp107, Ser192, Gly330, Ser331, Lys361	Pro180, Lys181, Glu191, Asp329
NS5	SURAMIN	- 12	7	2.80	Gly536, Thr539, Asn609, Ser661, Asp663, His798	Gln350, Phe354, Val358, Ala535, Asp538, Gln597, Arg598, Ser600, Thr605, Tyr606, Gly662, Lys689, Cys709, Trp795, Ser796, Ile797
	GALLIC ACID	- 5.3	4	2.87	Trp477, Lys578, Gly601	Val450, Arg481, Lys575, Val576, Val577, Gly599, Gln602
	NARINGIN	- 8.4	5	2.80	Trp477, Gln597, Gly601, Gln602	Trp302, Gln350, Phe354, Val358, Val450, Arg481, Asp538, Thr539, Lys578, Val576, Val577, Val579, Arg598, Ser600
	QUERCETIN	- 7.8	1	2.92	Trp477	Val353, Phe354, Val358, Arg481, Lys577, Val577, Val579, Gly599, Gly601, Gln602
	CATECHIN	- 7.2	6	2.73	Ser600, Tyr606, Asn609, Ser661, Asp663, His798	Thr605, Gly662, Ile797
	HESPERIDIN	- 8.8	7	2.86	Asp533, Asp538, Asn609, Asp663, Lys689, Arg729, His798	Tyr606, Gly662, Asp664, Cys709, Ser710, His711, Thr794, Ser796, Ile797

## Discussion

At present, due to the lack of a specific antiviral drug, different strategies are being used to control symptoms in patients with dengue, such as the administration of fluids and corticosteroids and the transfusion of blood derivatives [46]. Considering the agenda of research priorities proposed by WHO as well as the biodiversity of plants in Colombia, we searched for antivirals derived from natural products that could be evaluated in preclinical/clinical studies in the medium/long term.

This study first determined the selectivity of 12 ethanolic extracts derived from plants collected in the Colombian Caribbean coast. According to the SI values, the extracts were classified into four groups (Table 1). The group of nonselective extracts (SI < 2.0) comprised three plants. In contrast to our results, extracts of *C. citratus* have been reported to inhibit DENV infection by less than 50% [47]. The differences in the results could be due to the serotype tested as well as the low specificity of the technique used in the previous report (a qualitative technique based on the cytopathic effects produced by DENV-1). An inhibition of less than 50% could indicate low selectivity, which would be consistent with our results. Antiviral activity has not been reported for the other plants in Group A, but antioxidant activity has been reported for extracts from *D. inconstans* [48], and antitumor [49] and antifungal activities [50] have been documented for extracts from *Ch. icaco*. For the three plants in Group B (low selectivity, SI ≥ 2.0 and < 5), this study is the first to report anti-DENV activity. However, antitumor activity has been reported for *C. malambo* [51] and *A. cumanensis* [52], and lymphocyte proliferative activity has been documented for extracts from *T. hirta* [53]. The third group included plants whose extracts had moderate selectivity (SI ≥ 5 and < 10) and included *Ch. ambrosioides, C. platanifolia, M. charantia,* and *M. americana*. Previous reports of antibacterial and insecticidal [54], antileishmanial [55], and antimalarial activities have been described for *C. ambrosioides* L. [56]. Extracts of this plant have also been reported to increase nitric oxide production [57], which would favor its possible antiviral activity as increases in nitric oxide are known to lead to a decrease in DENV-2 replication [58]. For *M. charantia*, anti-DENV-1 activity has been reported for both extracts [47] and MAP30 protein derived from the plant [59]. Anti-DENV-2 activity has been described for two coumarins derived from *M. americana* [36]. This report is the first to document bioactivity for *C. platanifolia*. It is important to clarify that we decide to evaluate extracts from this plant, because the results from an ethnobotanical survey conducted in the city of Cartagena (Colombia) in 2009. Finally, the fourth group included the *P. guajava* extract*,* which was the only one with high selectivity (SI ≥ 10).

*P. guajava* (which belongs to the Myrtaceae family and Myrtoideae subfamily) is a tree that grows in tropical America and tropical & subtropical regions of Asia. It is recognized mainly for its fruit, the guava [60]. The bioactivities of some parts of the *P. guajava* have been described for several viral infections. For example, extract from *P. guajava* leaves inhibits approximately 50% of human and simian rotavirus infections in vitro [61], and aqueous and methanol extracts can inhibit murine leukemia virus retrotranscriptase at percentages greater than 50% [62]. In our study, the extract of this plant proved to be highly selective (SI = 128.2) with a percentage of inhibition greater than 90% (data not shown). The inhibitory effect of leave extracts from *P.guajava* in culture infected with DENV has been reported previously showed a IS of 153.18 μg/mL (very similar to our results) [63]. By other hand, some studies have shown that extracts derived from *P. guajava* are effective against diabetes mellitus due to its antihyperglycemic activity via inhibition the enzyme α-glucosidase [64].

Accordingly, the anti-DENV effect of the *P. guajava* extract could be linked to the inhibition of this enzyme, as it has been described as essential for the correct folding of viral glycoproteins and for virion assembly [65] during the replication cycle. Moreover, it has been reported that other fractions derived from *P. guajava* can have benefits in patients infected with Dengue. For example, in culture of HepG2 cells the trombinol, a bioactive fraction of *Psidium guajava,* induced the thrombopoietin production, and the authors postulate that this production could be considered as an alternative treatment in patients infected with dengue [66].

In this study, five fractions were isolated from an ethanolic extract of *P. guajava* bark, which showed a high content of tannins, alkaloids, and flavonoids (Table 2), in accord with the phytochemical composition described by other authors for this plant [67]. Only four of the five fractions showed selectivity, with the most selective being *Pg*-YP-I-22C (SI = 35.4). This value was almost four times lower than that obtained with the crude ethanolicic extract (SI = 128.2), which could indicate that the greater effectiveness of the extract may be due to synergy.

From the most selective fraction (*Pg-*YP-I-22C), five compounds were isolated and identified (Table 3 and Fig. 3). The first of these, gallic acid, is a polyphenolic compound [68] with diverse biological activity. Our results showed that gallic acid significantly inhibited viral activity via both the pre- and post-treatment strategies (percentages of inhibition greater than 50%). Previously, a cocktail (containing several compounds including gallic acid) derived from plants in the Phyllanthaceae family was shown to have an antiviral effect against DENV [69], unfortunately our results cannot be compared with those previously reported, as the effect of one compound alone is not comparable to that produced by the synergy of several compounds. However, recently has been reported that the isobutyl gallate (a gallic acid derivative) is an antiviral against DENV-2, with a SI = 25.6 in Huh 7 cells quite similar to our results in VERO cells (SI = 21.1) [70]. The second compound, naringin, is also a flavonoid with diverse biological activity [71]. In our study, naringin significantly inhibited DENV-2 infection only it was added to cells after viral inoculation (post-treatment). The anti-DENV activity of naringin has been previously demonstrated [72], but such inhibition occurred only when viral inoculation of the cells was performed in the presence of the compound (a method we did not use, thus making the results incomparable). This previous report did not investigate viral inhibition when the treatment was performed after inoculation, in contrast with our results. However, it is important to note that the concentrations we used were higher than those previously reported without being cytotoxic and that our SI (SI = 13.5) was ten times higher than that previously reported (SI = 1.3). The third compound, quercetin, is another flavonoid with diverse biological activity [73], including anti-DENV activity [72]. Although our results agree with those previously reported, we emphasize that the previously demonstrated selectivity (SI = 7.5) is much lower than that reported by us (SI = 34.3), which may be because we tested a higher concentration than that used previously. Quercetin has been postulated to directly inhibit the viral NS3 protein (a protein with multiple roles in DENV replication) [74], and it could interrupt virus entry by inhibiting fusion [75], making this compound a very promising antiviral. Catechin, the fourth compound isolated, is a polyphenol that is mainly derived from green tea [76]. Of the five compounds identified in this study, catechin is the one that induced the best viral inhibition when added before (100% inhibition) or after inoculation (91.8%). Recent molecular docking studies have shown that catechin has a high binding affinity for the NS4B protein of DENV [77], which is an important protein in the formation of the viral replication complex (together with NS4A) in the endoplasmic reticulum of host cells [78]. Finally, the fifth compound, hesperidin, is a flavonoid [79] of which no antiviral effect has been reported, agreeing with our results. It was the only nonselective compound (SI = 1.8) and did not inhibit infection in any of the experimental strategies used.

In addition to the in vitro studies*,* we used computational tools to explore the possible mechanisms involved in viral inhibition. For this, the E and NS5 proteins were selected as targets for the compounds tested, as in vitro inhibition was found for pre- and post-entry steps and their three-dimensional structures were available for in silico tests*.* Using computational methods, new antiviral molecules targeting the NS3 protease have already been reported that, when evaluated in vitro*,* inhibited DENV-2 infection up to 1 log PFU/mL [80], demonstrating the power of these methods in facilitating the search for new antiviral molecules.

In our study, for the E protein, the structure used corresponded to domain III of DENV-2; however, the crystal structure probably corresponds to the mature protein, as it was co-crystallized with the variable portion of monoclonal antibody 4E11 [81]. Nonetheless, based on the in vitro experiments, the molecules tested might influence the immature protein, possibly affecting the interaction by molecular docking. For the NS5 protein, even though the crystal structure corresponds to the catalytic domain [82], the binding pocket was defined using computational tools; thus, the docking energy values could vary. However, the negative docking energy values suggest that all the interactions were favorable [83]. Notably, the interaction of suramin with both proteins was favorable, but the best docking energy was found for its interaction with NS5 (− 12.0 kcal/mol), which was mediated by six hydrogen bonds (Fig. 6). This result is consistent with the data from the in vitro experiments in which better inhibition was observed for this compound via the post-treatment strategy (Fig. 4), suggesting that this compound acts on viral polymerase.

The docking energy values depend not only on the binding site but also on the virus type, target protein, and ligand source. A consensus model of the NS5 protein from four DENV serotypes was used previously against natural compounds obtained from the PubChem database22 and the SuperNatural II database2, yielding binding energies of < − 10.5 kcal/mol [84]; conversely, the compounds tested herein were obtained by conventional bioprospecting methods. The hydrogen bonds and hydrophobic interactions between the target protein and ligands determine the molecular docking score [85], and the distance between the atoms that are part of the bond is of great biological importance, especially if they range between 2.5 and 3.5 Å, as those with small distances are more relevant [83]. Such is the case of the hesperidin-E interaction, which has 11 theoretical hydrogen bonds compared to the four theoretical hydrogen bonds formed in the catechin-E interaction (Table 4).However, the shortest distance between the atoms forming a hydrogen bond in the protein-ligand pairs was obtained in the catechin-E interaction (2.70 Å). The distance between atoms was also the lowest (2.73 Å) in the catechin-NS5 interaction compared to those of the other interactions evaluated. Similar studies have reported that catechin can interact with other viral proteins such as NS4B from DENV-2 with negative docking scores ​​(− 4.06 kcal/mol), which suggests that this interaction would be viable [77]. Other factors that can affect the docking score are ligand size and the number of atoms forming bonds. Consequently, smaller ligands such as gallic acid, quercetin, and catechin have lower docking scores and lower numbers of hydrophobic interactions compared to those of naringin and hesperidin (Table 4).

In spite of the importance of ENV and NS5 proteins for viral replication, well-conserved non-structural proteins such as NS1, NS3/NS2B have been also evaluated against phytochemicals reported in literature, and the evaluated interactions were favorable with scores ranging from − 6.71 to − 32.24 Kcal/mol according to MOE Dock tool [86] Similarly, NS3 was evaluated against the compounds derived from the medicinal plant *Vetiveria zizanioides,* and one compound showed favorable interactions according to the scores obtained with Surflex-dock, mediated by Hydrogen bonds with 2.72 Å being the lowest distance found mediating this interactions [87].

Although bioprospecting has been replaced by more targeted tools, it is still useful for identifying antiviral compounds, as we have shown in this study. Additionally, combining in vitro and in silico tests allowed us not only to identify promising antivirals but also to suggest their possible mechanisms of action.

## Conclusions

Our results showed that four compounds derived from *P. guajava* highly selectively inhibited DENV-2 replication. Catechin is the most promising compound with viral inhibition percentages of greater than 90% in the two different experimental strategies. Studies are in progress that will allow us to elucidate the antiviral mechanisms of these compounds.

## Data Availability

All the materials are described within the manuscript. Moreover the most relevant data are contained within the manuscript too; and the raw data are available to other researchers upon request.

## Abbreviations

CC_50_: 50% cytotoxicity concentration
DENV: Dengue virus
EC_50_: 50% effective concentration
PBS: Phosphate-buffered saline
SI: Selectivity Index
